# Human HERC5 restricts an early stage of HIV-1 assembly by a mechanism correlating with the ISGylation of Gag

**DOI:** 10.1186/1742-4690-8-95

**Published:** 2011-11-17

**Authors:** Matthew W Woods, Jenna N Kelly, Clayton J Hattlmann, Jessica GK Tong, Li S Xu, Macon D Coleman, Graeme R Quest, James R Smiley, Stephen D Barr

**Affiliations:** 1 The University of Western Ontario, Schulich School of Medicine and Dentistry, Center for Human Immunology, Department of Microbiology and Immunology, Dental Sciences Building Room 3006b, London, Ontario, Canada; 2 University of Alberta, Alberta Institute for Viral Immunology, Faculty of Medicine & Dentistry Department of Medical Microbiology and Immunology, 6-32 Heritage Medical Research Building. T6G 2S2, Edmonton, Alberta, Canada

**Keywords:** HERC5, ISG15, E3 ligase, Gag assembly, cellular restriction factor, interferon, HIV, MLV

## Abstract

**Background:**

The identification and characterization of several interferon (IFN)-induced cellular HIV-1 restriction factors, defined as host cellular proteins or factors that restrict or inhibit the HIV-1 life cycle, have provided insight into the IFN response towards HIV-1 infection and identified new therapeutic targets for HIV-1 infection. To further characterize the mechanism underlying restriction of the late stages of HIV-1 replication, we assessed the ability of IFNbeta-induced genes to restrict HIV-1 Gag particle production and have identified a potentially novel host factor called HECT domain and RCC1-like domain-containing protein 5 (HERC5) that blocks a unique late stage of the HIV-1 life cycle.

**Results:**

HERC5 inhibited the replication of HIV-1 over multiple rounds of infection and was found to target a late stage of HIV-1 particle production. The E3 ligase activity of HERC5 was required for blocking HIV-1 Gag particle production and correlated with the post-translational modification of Gag with ISG15. HERC5 interacted with HIV-1 Gag and did not alter trafficking of HIV-1 Gag to the plasma membrane. Electron microscopy revealed that the assembly of HIV-1 Gag particles was arrested at the plasma membrane, at an early stage of assembly. The mechanism of HERC5-induced restriction of HIV-1 particle production is distinct from the mechanism underlying HIV-1 restriction by the expression of ISG15 alone, which acts at a later step in particle release. Moreover, HERC5 restricted murine leukemia virus (MLV) Gag particle production, showing that HERC5 is effective in restricting Gag particle production of an evolutionarily divergent retrovirus.

**Conclusions:**

HERC5 represents a potential new host factor that blocks an early stage of retroviral Gag particle assembly. With no apparent HIV-1 protein that directly counteracts it, HERC5 may represent a new candidate for HIV/AIDS therapy.

## Background

Several IFN-induced proteins have been identified as correlates of HIV-1 infection and disease progression [1-4]. A few of these factors have already been shown to exhibit antiviral activity towards HIV-1, such as apolipoprotein B mRNA-editing enzyme catalytic polypeptide (APOBEC) 3G, bone marrow stromal antigen 2 (BST2)/tetherin and tripartite motif-containing protein 22 (TRIM22) (reviewed in [5]). To identify and characterize additional potential host factors, we surveyed IFNbeta (IFNβ)-induced genes from two previous studies [6,7] for potential anti-HIV-1 activity and identified HERC5 as a new host factor that may block a late stage of the HIV-1 life cycle.

The *herc5 *gene is one of six human *herc *family genes located on chromosome 4 and flanked by *herc6 *and *herc3*. The HERC5 protein is 1024 amino acids in length and contains a Regulator of Chromosome Condensation 1 (RCC1)-like domain (RLD) at its amino-terminus, followed by a unique Spacer region that does not share homology with any known protein, and a HECT domain at the carboxyl-terminus [8-10]. The function of the RLD domain is unknown; however, it appears to be important, but not essential, for the conjugation of ISG15 to cellular proteins [6]. Also, the predicted tertiary structure of the RLD closely resembles the crystal structure of the human RCC1 protein (reviewed in [11,12]). The Spacer region contains numerous α-helices, and its function is unknown. The HECT domain is typically found in E3 ligases, which operate in conjunction with unique E1 "activating" and E2 "conjugating" enzymes to transfer ubiquitin or ubiquitin-like proteins, such as ISG15, to specific cellular substrates.

HERC5 is the main E3 ligase for the conjugation of ISG15 to proteins in human cells and works together with the E1 activating protein Ube1L as well as the E2 conjugating protein UbcH8 to conjugate ISG15 onto target proteins [6,13,14]. Cysteine 994 of the HERC5 HECT domain is essential for this E3 ligase activity of HERC5 [13]. Two reports have also identified HERC5 as an E3 ubiquitin ligase [15,16]. HERC5 is ubiquitously expressed with the highest levels of expression observed in the testis. HERC5 is up-regulated in a variety of primary cells and immortalized cell lines by interferon, lipopolysaccharide, tumor necrosis factor α, and interleukin-1β [6,13,16]. Moreover, HERC5 broadly targets newly synthesized proteins for ISG15 conjugation, and many endogenous targets of HERC5 have been identified that function in a variety of cellular pathways including RNA splicing, chromatin remodeling/polII transcription, cytoskeleton organization and regulation, stress responses, translation, glycolysis, interferon signaling and antiviral responses [17-22]. Here, we show that HERC5 inhibits HIV-1 replication by targeting a unique step of HIV-1 particle assembly at the plasma membrane.

## Results

### HERC5 inhibits HIV-1 particle production

**Figure 1:**
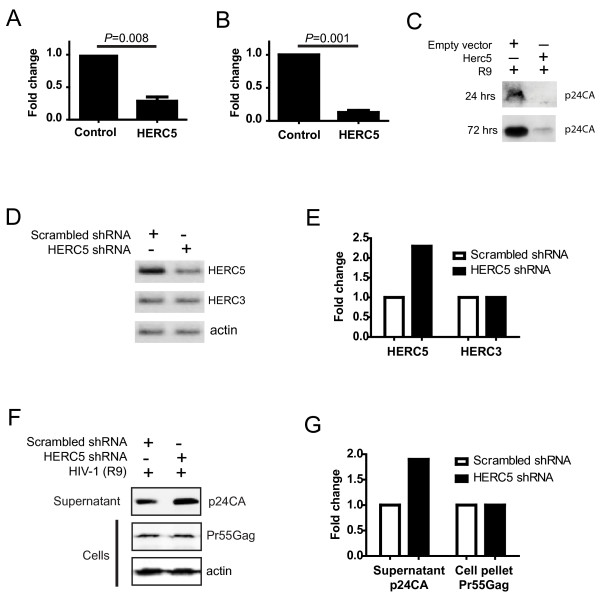
**HERC5 restricts a late stage of HIV-1 replication**. **A**, Inhibition of HIV-1 particle production. 293T cells were co-transfected with pR9 and either empty plasmid or pHERC5. Twenty-four hours after transfection, infectious HIV-1 virions released into the supernatant were quantified using GHOST(3) indicator cells. **B**, Inhibition of HIV-1 replication. HOS-CD4/CXCR4 cells were co-transfected with plasmids encoding a replication-competent HIV-1 provirus (R9) and HERC5 or an empty vector control. HIV-1 virions released into the supernatant were pelleted 72 hours after transfection and analyzed by Western blotting using anti-p24CA antibody. p24CA levels were quantified densitometrically. **C**, Representative Western blot using anti-p24CA of HIV-1 particles released into the supernatant. Data shown are the average of at least two independent experiments performed in triplicate. **D-G**, 293T cells were co-transfected with pLKO.1/scrambled_shRNA _or pLKO.1/HERC5_shRNA _and pR9. Twenty-four hours after transfection, HIV-1 particles released into the supernatant, cellular RNA and cellular protein were isolated. HERC5, HERC3 and β-actin RNA were detected by reverse transcription polymerase chain reaction (RT-PCR) (**D**) and quantified densitometrically (**E**). HIV-1 particles released into the supernatant and intracellular Pr55Gag protein were detected by Western blotting using anti-p24CA (**F**) and quantified densitometrically (**G**). Data shown are representative of at least two independent experiments.

HERC5 is the main cellular E3 ligase that conjugates ISG15 to proteins and can target newly synthesized proteins, including foreign proteins [6,13,21]. Therefore, we tested the ability of HERC5 to restrict the release of newly made infectious HIV-1 particles. We co-transfected 293T cells with empty vector or plasmids encoding replication-competent HIV-1 (pR9) and HERC5. Cells expressing HERC5 released 4.0-fold less infectious virus than the control cells after a single round of replication (*P *= 0.008, paired t test) (Figure 1A). Similar results were also obtained in HOS-CD4/CXCR4 cells, which support robust HIV-1 replication. HOS-CD4/CXCR4 cells expressing HERC5 released 10.8-fold less infectious HIV-1 particles into the supernatant after multiple rounds of replication (*P *= 0.001, paired t test) (Figure 1B). Similar levels of inhibition of HIV-1 particle release after single and multiple rounds of replication were observed by Western blot analysis (Figure 1C). There was no significant difference in the viability of cells expressing HERC5 compared to the control cells (90.2% ± 4.4% SD versus 92.0% ± 4.3% SD respectively; *P *> 0.05, paired t test).

To assess the impact of reduced levels of HERC5 on HIV-1 particle production, we depleted HERC5 using short hairpin ribonucleic acid (shRNA) and measured the amount of HIV-1 particles released into the supernatant. 293T cells, which express basal levels of HERC5, were transiently co-transfected for 24 hours with either pLKO.1/scrambled_shRNA _or pLKO.1/HERC5_shRNA _and pR9. Cells expressing HERC5 shRNA exhibited 2.3-fold less HERC5 RNA than the control cells expressing scrambled shRNA (Figure 1D and 1E). As a control for specificity, RNA levels of the related *herc3 *gene were tested and found to be unaffected by the scrambled or HERC5 shRNA (Figure 1D and 1E). Accumulation of HIV-1 particles in the supernatant and cell pellets was monitored by Western blot using anti-p24CA (Figure 1F). Cells knocked down for HERC5 expression released 1.9-fold more HIV-1 particles into the supernatant compared to the control cells (Figure 1F and 1G). No substantial change in the intracellular Pr55Gag protein level was detected. Similar results were observed in HeLa cells, which also express basal levels of HERC5 (data not shown).

### HERC5 blocks HIV-1 Gag particle production and this block correlates with the ISGylation of Gag

Gag is the major structural polyprotein of HIV-1 that drives virion formation and can assemble and bud from cells in the absence of all other HIV-1 proteins as Gag-only particles [23]. Therefore, we asked if HERC5 could conjugate ISG15 to Gag, and in so doing, interfere with Gag particle production. To test this, we used a single-cycle HIV-1 Gag-only particle release assay in U2OS cells, which do not express HERC5 mRNA basally or after IFNβ treatment, thereby providing a HERC5 null background (reference [14] and data not shown). The Gag-only particle release assay involved the co-transfection of plasmids encoding codon-optimized HIV-1 Gag with or without HERC5 and the measurement of the Gag-only particles released into the supernatant by quantitative Western blotting. To enhance detection of Gag modified with ISG15, plasmids encoding histidine-tagged ISG15, Ube1L and UbcH8 (referred to hereafter as the conjugation system (CS)), were included in the transfections.

**Figure 2:**
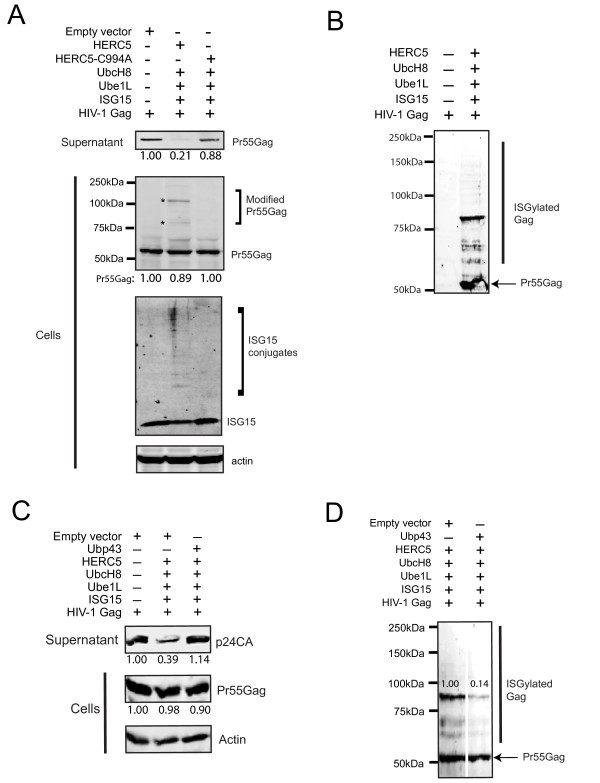
**HERC5 restricts HIV-1 Gag particle production by a mechanism dependent on its E3 ligase activity**. **A**, U2OS cells were co-transfected with pGag, pUbe1L, pUbcH8, pMyc-ISG15 and either empty plasmid, pHERC5 or pHERC5-C994A. Gag particles released into the supernatant and intracellular Gag protein expression were analyzed after 24 hours by quantitative Western blotting using anti-p24CA and anti-β-actin as a loading control. The same blot was stripped and tested for ISG15 conjugates using anti-myc (bottom blot). **B**, Cells were co-transfected with empty plasmid or pHERC5 and pUbe1L, pUbcH8, pHis-ISG15 and pGag. Cells were lysed 72 hours later and histidine-tagged proteins were purified using nickel agarose. Purified proteins were resolved using SDS-PAGE and subjected to Western blotting using anti-p24CA. **C**, Cells were co-transfected with empty vector or pHERC5, pUbe1L, pUbcH8, pHis-ISG15 and pGag with or without pUbp43. Gag particles released into the supernatant and intracellular Gag protein expression were analyzed after 72 hours by quantitative Western blotting using anti-p24CA or anti-β-actin. **D**, In a similar transfection as in C, histidine-tagged proteins were purified from cell extracts using nickel agarose and subjected to Western blotting using anti-p24CA. The two lanes were digitally separated from the same image. Numerical values on the blots display the densitometric quantification of the specified bands. All Western blots shown are representative of at least two independent experiments.

As shown in Figure 2A, Western blot analysis revealed that cells expressing HERC5 + CS released substantially less Gag-only particles into the supernatant than the control cells. In contrast, cells expressing HERC5-C994A (defective for its E3 ligase activity) + CS failed to inhibit the release of Gag-only particles. Western blot analysis of Gag protein expression in cell lysates revealed similar levels of Gag (55 kDa) in all samples. Notably, more slowly migrating Gag species (~85 kDa and ~110 kDa) were detected in cells expressing HERC5 + CS. These slowly migrating Gag bands were not present in the HERC5-C994A + CS samples, suggesting that the E3 ligase activity of HERC5 was required for generating the more slowly migrating Gag species (Figure 2A). Western blot analysis of the cell lysates revealed the presence of ISG15 conjugates in cells transfected with HERC5 + CS, but not cells transfected with empty vector or HERC5-C994A, as previously shown [13]. The sizes of these slowly migrating Gag species are consistent with the conjugation of two or more ISG15 proteins (one ISG15 = ~15 kDa), although non-linear decreases in the electrophoretic mobility of proteins conjugated with ISG15 in SDS-PAGE gels have been previously reported [24,25]. The identities of the slowly migrating Gag species were confirmed to be Gag after immunoprecipitation and protein identification using matrix-assisted laser desorption/ionization mass spectrometry (MALDI-MS). There was no significant difference in the viability of cells expressing HERC5 + CS compared to the control cells (92.0% ± 4.3% SD versus 90.2% ± 4.4% SD respectively; *P *> 0.05, paired t test).

To determine if the more slowly migrating bands of Gag contained conjugates of ISG15, we co-expressed Gag and HERC5 with histidine-tagged ISG15 and the CS for 72 hours and then incubated the cell lysate with nickel agarose under non-denaturing conditions. Western blot analysis of the purified proteins using anti-p24CA revealed the presence of several species of Gag ranging from ~55 kDa to ~200 kDa in size (Figure 2B), including the ~85 kDa and ~110 kDa bands detected in the whole cell lysate (Figure 2A). Notably, when the ISG15ylated Gag species was purified under non-denaturing conditions, a substantial amount of 55 kDa Gag species was co-purified, suggesting that Gag species conjugated with ISG15 can interact with the 55 kDa unmodified Gag (Figure 2B). It is unknown why the ~85 kDa Gag species was more abundant than the ~110 kDa species after purification with the nickel agarose (compared to the levels observed in the cell lysate (Figure 2A). We have noticed that the ~110 kDa species is more abundant if Gag is purified 48 hours after transfection instead of after 72 hours, possibly indicating that some of the modifications are labile.

To assess the importance of ISG15ylation in restricting Gag particle release, we co-expressed HERC5 + CS with the ISG15-specific deconjugating enzyme Ubp43 and assessed whether Ubp43 could rescue restriction imposed by HERC5 [26]. Quantitative Western blot analysis of Gag particles released into the supernatant showed that Ubp43 abolished HERC5-induced restriction, despite the presence of similar levels of intracellular Gag (Figure 2C). The rescue of Gag particle release by Ubp43 correlated with a 7.2-fold reduction in intracellular ISG15ylated Gag (Figure 2D).

### Intracellular localization of HERC5 and HIV-1 Gag

**Figure 3:**
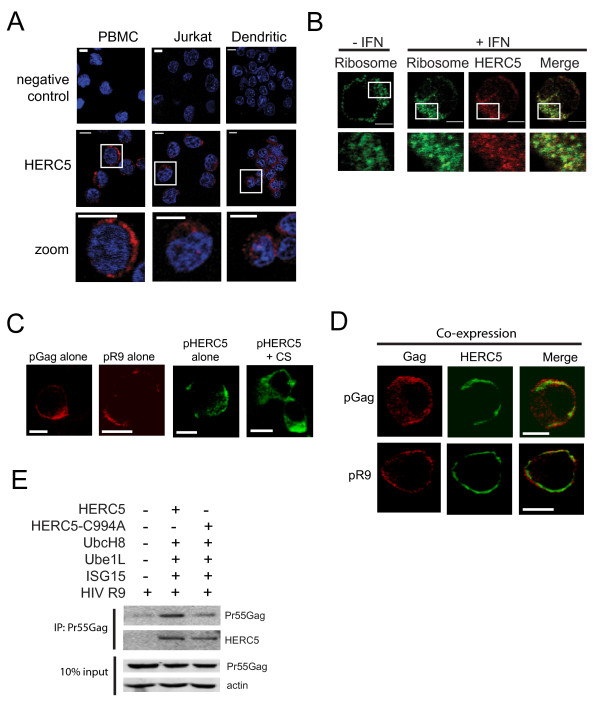
**Intracellular localization of IFNβ-treated HERC5 and HIV-1 Gag**. **A**, Localization of endogenous HERC5 (red) in IFNβ-treated (500 units/ml for 16 hours) PBMCs (scale bar = 5 μm), Jurkat cells (scale bar = 5 μm), and murine dendritic cells (scale bar = 10 μm). Images shown are representative of at least 2 independent experiments. **B**, Localization of endogenous ribosomes (green) and HERC5 (red) in control Jurkat cells or IFNβ-treated Jurkat cells (scale bar = 5 μm). Localization of Gag alone (red) or HERC5 (green) +/- CS (**C**), or co-expression of Gag and HERC5 + CS (**D**) in U2OS cells 48 hours after transfection. Scale bars = 20 μm. Images shown are representative of at least 3 independent experiments. **E**, HeLa cells were co-transfected with pR9, pUbe1L, pUbcH8, and pMyc-ISG15 with or without pFlag-HERC5 or pFlag-HERC5-C994A. Gag was immunoprecipitated under non-denaturing conditions using anti-p24CA. Precipitated proteins were resolved using SDS-PAGE and subjected to Western blotting using anti-Flag or anti-p24CA. 10% of the input lysates was subjected to Western blotting using anti-p24CA and anti-β-actin on the same blot. HERC5 protein was undetectable in the 10% input lysates.

Confocal immunofluorescence microscopy of IFNβ-treated PBMCs, human Jurkat cells, and immortalized murine mature dendritic cells (DC2.4) revealed that HERC5 localized to the cytoplasm in punctate bodies in each of the cell types (Figure 3A), consistent with the pattern of localization observed for HERC5 in human mammary epithelial cells [15]. Recently, it was shown biochemically in HeLa cells, but not microscopically, that HERC5 co-fractionated with polysomes due to an association with the 60S ribosomal subunit [21]. Consistent with this report, we observed that 59.9% ± 0.12% SD of HERC5 co-localized with polyribosomes in IFNβ-treated Jurkat cells using confocal immunofluorescence microscopy (mean Pearson's coefficient = 0.855; *n *= 10) (Figure 3B). Similar observations were made in HOS-CD4/CXCR4 cells where 61.7% ± 0.13% SD of IFNβ-induced HERC5 co-localized with ribosomes (mean Pearson's coefficient = 0.863; *n *= 10).

To determine if HERC5 and HIV-1 Gag co-localize in cells, we co-transfected plasmids encoding Gag-only (pGag) or replication-competent HIV-1 (pR9) with and without HERC5 + CS. Forty-eight hours after transfection, the localization of Gag and HERC5 protein was assessed in U2OS cells by examining optical slices through the center of cells. Gag expressed in the absence of HERC5 exhibited predominantly punctate fluorescence at the plasma membrane, consistent with the location of assembly and budding of Gag particles (Figure 3C). HERC5 expressed in the presence or absence of the conjugation system localized predominantly in the cytoplasm in punctate bodies (Figure 3C). When Gag was co-expressed with HERC5 + CS, Gag localized predominantly at the plasma membrane (Figure 3D). Interestingly, a substantial amount of HERC5 accumulated near the plasma membrane where 65.2% ± 0.21% SD of Gag expressed from pR9 co-localized with HERC5 (mean Pearson's coefficient = 0.324; n = 21) and 53.1% ± 0.16 SD of Gag expressed from pGag co-localized with HERC5 (mean Pearson's coefficient = 0.336; n = 15).

Since it was recently shown that HERC5 can conjugate ISG15 to exogenously expressed foreign proteins [21], we asked whether we could detect an interaction between flag-tagged HERC5 and HIV-1 Gag biochemically. Flag-tagged HERC5 + CS or flag-tagged HERC5-C994A + CS were co-expressed with replication-competent HIV-1 (R9) in HeLa cells. Forty-eight hours post-transfection, cells were lysed under non-denaturing conditions and subjected to co-immunoprecipitation using anti-p24CA. Western blot analysis of the precipitated proteins revealed that both flag-tagged HERC5 and HERC5-C994A co-precipitated with HIV-1 Gag (Figure 3E). Despite similar input levels of intracellular Gag, more Gag was consistently immunoprecipitated in the presence of HERC5 compared to the control and HERC5-C994A. Similar results were obtained with 293T cells (data not shown).

### HERC5 inhibits an early stage of Gag assembly

**Figure 4:**
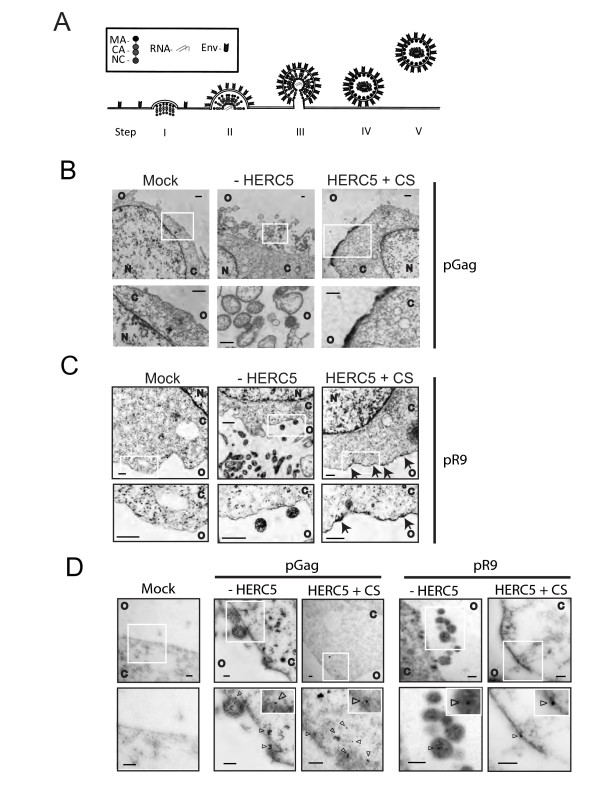
**HERC5 inhibits an early stage of Gag assembly at the plasma membrane**. **A**, Schematic diagram of HIV-1 Gag assembly, budding and release at the plasma membrane. step I, Gag accumulates at the plasma membrane; step II, distortion of plasma membrane curvature; step III, increased distortion of the plasma membrane and formation of the "lollipop structure;" step IV, virion detaches its membrane from the host cell, undergoes proteolytic processing of Gag and remains closely associated with the host cell; step V virion distances itself from the host cell. **B**, Transmission electron micrographs of U2OS cells that were mock transfected ("mock"), transfected with pGag and empty vector ("-HERC5"), or co-transfected with pGag and pHERC5 + CS ("HERC5 + CS"). Scale bars = 500 nm. **C**, Transmission electron micrographs of U2OS cells that were mock transfected ("mock"), transfected with pR9 and empty vector ("-HERC5"), or co-transfected with pR9 and pHERC5 + CS ("HERC5 + CS"). Scale bars = 300 nm. **D**, Transmission electron micrographs of U2OS cells that were transfected as described in parts B and C showing immunogold labeling of p24CA (Gag). Gold particles are 10 nm in size. Bottom panels of the micrographs are zoomed images of the white boxes in the upper panels. Scale bars = 100 nm. N, nucleus; C, cytoplasm; O, outside of cell.

We then utilized transmission electron microscopy (TEM) to examine HIV-1 Gag particles assembling at the plasma membrane in the presence or absence of HERC5. As normally seen phenotypically with TEM micrographs of negatively-stained samples, HIV-1 Gag particle assembly at the plasma membrane begins with the accumulation of Gag protein at the plasma membrane, which is visualized as small electron-dense regions at the plasma membrane often seen as small buds with the diameter of an immature Gag shell ~150-200 nm (step I). This is followed by the formation of more pronounced half-spherical particles protruding from the plasma membrane (step II), more extensive budding with nearly complete spherical particles that are still contiguous with the cytoplasm via a stalk-like ("lollipop") structure (step III), scission of viral and cellular membranes (step IV), and finally release and distancing of the HIV-1 Gag particle from the plasma membrane (step V) (Figure 4A).

To differentiate between the various stages of assembly, budding and release, we examined TEM images of mock transfected U2OS cells and cells transfected with plasmids encoding codon-optimized HIV-1 Gag with or without HERC5 + CS. U2OS cells were chosen because of their HERC5 null background and that HIV-1 particles assemble and bud at their plasma membrane normally. As shown in Figure 4B, the mock control cells exhibited a relatively unperturbed plasma membrane, the absence of electron-dense accumulations/patches and budding structures at the plasma membrane. In contrast, cells expressing Gag in the absence of HERC5 + CS exhibited substantial perturbations of the plasma membrane and budding Gag-only particles. The Gag-only particles appeared as enveloped, spherical particles that resembled immature HIV virions but more heterogeneous in size (up to ~500 nm in diameter). This observation is in agreement with previously published results [27]. Strikingly, cells expressing Gag in the presence of HERC5 + CS yielded substantially less Gag-only particles and exhibited pronounced electron-dense staining at the plasma membrane appearing in a polarized region of the plasma membrane or at regions of cell-cell-contact (Figure 4B).

To determine if the pronounced accumulation of Gag at the plasma membrane was due to gross over-expression from the cytomegalovirus (CMV) promoter-driven codon-optimized Gag (pGag), we performed a similar TEM experiment, except that we transfected a plasmid encoding replication-competent proviral HIV-1 (pR9). This plasmid expresses biologically relevant levels of wild type Gag (in addition to all other HIV-1 proteins) from the HIV-1 long terminal repeat promoter. As seen in Figure 4C, the plasma membrane of mock transfected cells exhibited a relatively unperturbed profile with no viral particles released from the plasma membrane, whereas cells expressing HIV-1 alone produced numerous predominantly homogeneous particles (~100-150 nm in diameter). Cells transfected with pR9 and pHERC5 + CS released substantially less HIV-1 particles compared to cells expressing R9 only. Notably, numerous cells exhibited electron-dense regions resembling early HIV-1 Gag assembly structures at the plasma membrane, with the absence of budded "lollipop" structures, extracellular virions, and intracellular virions. The electron dense patches accumulated in a polarized region of the plasma membrane or at regions of cell-cell contact. The electron-dense regions of the early budding structures are ~150-200 nm in diameter, which is consistent with the diameter of a budding immature Gag shell [28]. To confirm that Gag protein accumulated in HIV-1 Gag particles and electron dense regions at the plasma membrane, we performed immunogold-labeling using anti-p24CA. As shown in Figure 4D, Gag was observed to specifically localize in each of these regions. Quantification of immunogold localization on the electron microscopic thin sections from cells expressing HERC5 + CS and Gag-only or R9 showed that the gold labeling distribution was significantly different from a random distribution (Chi square analysis, *X^2 ^*= 314.8, df = 2, *P *< 0.0001) (Table 1) (see Methods for details).

**Table 1:** Quantification of 10 nm gold particle-labeled anti-p24CA in cells expressing HERC5+CS and HIV-1 Gag.

Region	Observed gold count, Go	Point count, P	Expected gold count, Ge	Go/Ge	Χ^2^	Χ^2 ^as %
**Particles+** **Plasma membrane**	118	189	29	4.0	270.3	85.9
**Cytoplasm+** **Nucleus**	80	875	135	0.6	22.5	7.1
**Non-particle+** **non-cell**	18	335	52	0.3	22.0	7.0
**TOTAL**	216	1399	216		314.8	100.0

For *Χ*^2 ^= 314.8 and df = 2, *P *< 0.0001 (*Χ*^2 ^analysis). The gold labeling distribution is significantly different from random. Only the particles/plasma membrane region (Go/Ge = 4.0, *Χ*^2 ^= 85.9% of total) meets the two criteria for being preferentially labeled ((Go/Ge) > 1 and *Χ*^2 ^value ≥ 10% of total).

### HERC5-induced restriction of Gag particle production is distinct from ISG15-only restriction

Three reports suggest that ectopic expression of the CS only (minus HERC5) restricts HIV-1 Gag particle budding and/or release [29-31]. Since HERC5 is the main enzyme responsible for the conjugation of ISG15 to proteins, we asked if HERC5 was the E3 ligase responsible for the restriction of Gag-only particle release induced by ISG15 expression. To address this question, we performed a single-cycle Gag-only release assay by co-expressing Gag + CS only in U2OS cells (which lack HERC5 expression). Consistent with the three previous published reports [29-31], we observed that the CS blocked Gag particle production without affecting the levels of intracellular Gag protein (data not shown).

**Figure 5:**
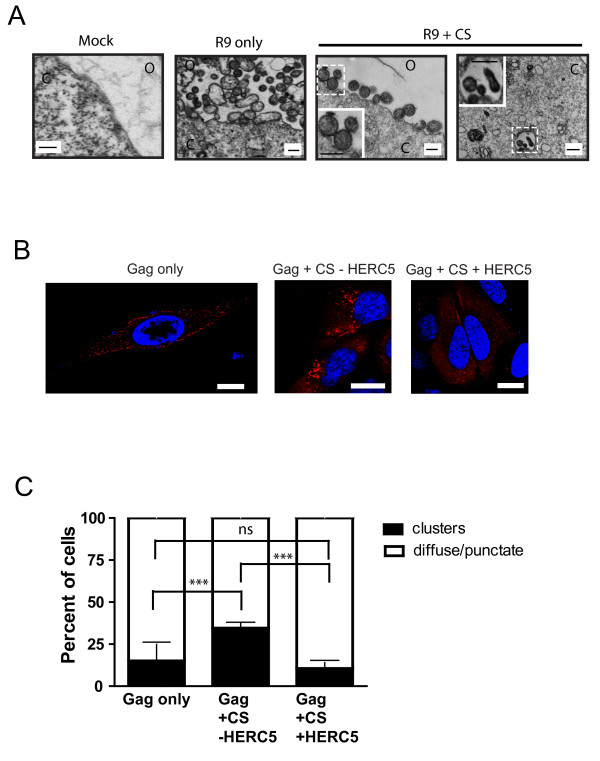
**ISG15 expressed in the absence of HERC5 causes retention of virions on cell surfaces and in intracellular compartments**. **A**, Transmission electron micrographs showing the plasma membrane and intracellular regions of U2OS cells that were mock transfected ("mock"), co-transfected with empty vector and pR9 ("R9 only"), or co-transfected with pR9 and CS only ("R9 + CS"). Insets show zoomed images of the dotted white boxes. Scale bars = 200 nm. **B**, Confocal immunofluorescence microscopy of U2OS cells mock transfected or co-transfected with pGag with or without HERC5, and the CS. Gag localization was detected using anti-p24CA (red) 48 hours post-transfection. Scale bars = 20 μm. **C**, Quantitative analysis of cells from part B containing punctate Gag at the plasma membrane and large intracellular clusters of Gag or intracellular diffuse/punctate Gag. Data represent the average of two independent experiments +/- SD (n = 400). ns, non-significant. ***, *P *< 0.0001, Fisher's exact test.

To determine if the inhibition of Gag particle production by the CS alone resembled the inhibition of Gag particle production induced by CS + HERC5, we examined TEM micrographs of cells expressing replication-competent HIV-1 (R9) + CS only. Analysis of cells expressing R9 + CS revealed an accumulation of apparently fully enveloped immature virions on the surface of the cell in addition to virions within intracellular compartments (Figure 5A). Virions found on the cell surface were closely associated with each other as doublet particles or in long chains. This phenotype is in contrast with that observed when HIV-1 is expressed in the presence of HERC5 + CS (Figure 4C).

We also assessed the intracellular localization of Gag in these cells by confocal immunofluorescence microscopy analysis. As shown in Figure 5B, control cells transfected with pGag + empty vector exhibited numerous punctate signals predominantly at the plasma membrane and regions of cell-cell contact, consistent with the site of Gag particle budding. Similar to the control cells, cells transfected with pGag + CS + pHERC5 exhibited an accumulation of Gag predominantly at the plasma membrane and regions of cell-cell contact. Interestingly, in cells transfected with pGag + CS, Gag accumulated at the plasma membrane and in the cytoplasm as large clusters (Figure 5B).

Quantitative analysis of cells co-expressing Gag + CS -HERC5 revealed that 34.5% ± 3.5% SD of the cells contained Gag localized in the cytoplasm as large clusters. This is significantly more than cells expressing Gag only or Gag + CS + HERC5 where 15.0% ± 9.8% SD and 10.5% ± 3.5% SD of the cells contained large clusters of intracellular Gag, respectively (*P *< 0.0001, Fisher's exact test) (Figure 5C). Taken together, these data suggest that the mechanism of inhibition of HIV-1 Gag particle production is different in cells expressing CS + HERC5 than in cells expressing CS only.

### HERC5 restricts MLV Gag-containing particle production

**Figure 6:**
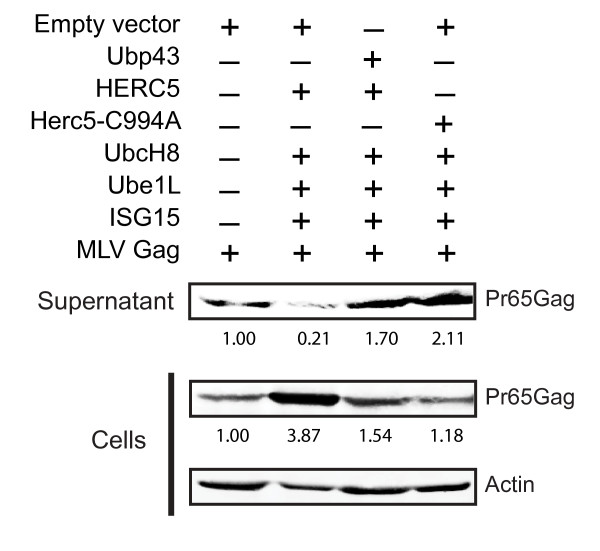
**HERC5 restricts MLV Gag particle production**. U2OS cells were co-transfected with pMLV-Gag and either empty plasmid, pHERC5 + CS, or pHERC5-C994A + CS with or without pUbp43. Gag particles released into the supernatant and intracellular Gag protein expression were analyzed by quantitative Western blotting using anti-MLV antisera or anti-β-actin 48 hours post-transfection. Numerical values on the blots display the densitometric quantification of the specified bands after normalization with β-actin levels.

To assess the specificity of HERC5 for retroviruses, we tested the ability of HERC5 to restrict Gag particle production of the simple retrovirus MLV. Using a similar Gag particle release assay as that used above for HIV-1 Gag, we found that HERC5 + CS markedly restricted the release of MLV Gag-containing particles into the supernatant (Figure 6). Similar to that with HIV-1, Ubp43 co-expression rescued HERC5-induced restriction of MLV Gag particle production. HERC5-C994A + CS did not restrict MLV Gag particle production suggesting that the E3 ligase activity is also required for MLV restriction as it is for HIV-1. In contrast with that of HIV-1, expression of HERC5 + CS caused a substantial accumulation of intracellular MLV Gag protein. This accumulation was prevented by co-expression with Ubp43 (Figure 6). Taken together, these data suggest that HERC5 restricts Gag particle production from an evolutionarily diverse retrovirus and that the mechanism involves the E3 ligase activity of HERC5 for ISG15 conjugation.

### HERC5 expression in HIV1-infected patients

**Figure 7:**
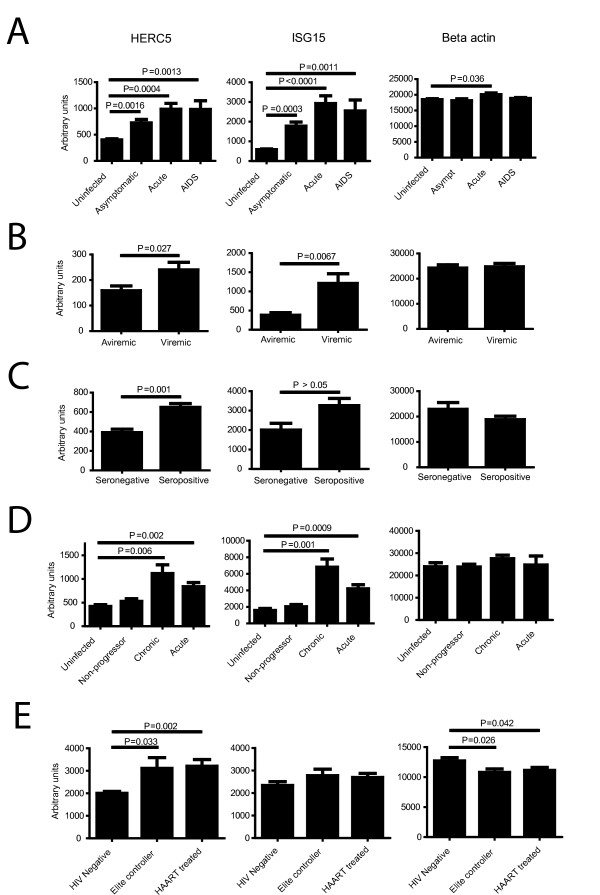
**HERC5 and ISG15 expression in HIV-1-infected patients**. The Gene Expression Omnibus database repository was searched to identify published transcriptional profiling datasets of HIV-1- infected patient samples. The average relative RNA expression levels of HERC5, ISG15 and beta actin were compared for each dataset. **A**, GSE16363 dataset from Li *et al. *(2009) comparing patients with asymptomatic and acute stages of AIDS and patients with AIDS. **B**, GDS2168 dataset from Tilton *et al. *(2006) comparing monocytes from viremic individuals after cessation of HAART and aviremic patients during HAART. **C**, GDS1449 dataset from Ockenhouse *et al. *(2005) comparing primary human PBMCs from seropositive patients compared to seronegative patients. **D**, GDS2649 dataset from Hyrcza *et al. *(2007) comparing CD4+ T cells from non-progressors, and acutely and chronically infected patients. **E**, GSE23879 dataset from Vigneault *et al. *(2011) comparing elite controllers and patients treated with HAART. Significance was calculated using Student's unpaired t test. *P *values < 0.05 were considered significant.

To assess the expression of *herc5 *in HIV-1-infected patients, we mined the Gene Expression Omnibus database repository http://www.ncbi.nlm.nih.gov/gds for published datasets containing gene expression profiles in HIV-1-infected individuals at various stages of disease progression. Transcriptional profiling data revealed that HERC5 and ISG15 RNA expression are significantly increased in lymphatic tissue during acute HIV-1 infection in patients with asymptomatic and acute stages of AIDS and patients with AIDS (Figure 7A) [2]. Significant increases in HERC5 and ISG15 RNA expression were observed in monocytes from viremic individuals after cessation of highly active antiretroviral therapy (HAART) compared to aviremic patients during HAART (Figure 7B) [3]. HERC5 and ISG15 expression is significantly increased in primary human PBMCs from seropositive patients compared to seronegative patients (Figure 7C) [4]. HERC5 and ISG15 expression is significantly increased in CD4+ T cells from acutely and chronically infected patients, but not in non-progressors (Figure 7D) [32]. HERC5, but not ISG15, is significantly increased in elite controllers and patients treated with HAART compared to HIV-1 negative patients (Figure 7E) [33]. Furthermore, HERC5 and ISG15 were identified as correlates of protection from HIV-1 infection in controllers compared to non-controllers (see reference [1]). In all datasets, there was no significant increase in the expression of beta actin, except in patients in the acute stages of AIDS, where a modest increase was observed (Figure 7A). Taken together, these data show that HERC5 expression is significantly increased in patients in the acute and chronic stages of infection and in patients in stages of long-term control of infection.

## Discussion

Here we have identified HERC5 as an IFN-induced protein that is able to block an early step of HIV-1 particle assembly at the plasma membrane. HERC5 and Gag proteins were found to associate with each other *in vitro *and to co-localize within cells. These interactions correlated with the modification of Gag protein with ISG15. HERC5 did not alter the trafficking of Gag to the plasma membrane; however, microscopic analysis revealed that Gag assembly was blocked at an early stage. HERC5 was also able to restrict the release of MLV Gag, showing that HERC5 can restrict an evolutionarily diverse retrovirus.

The mammalian Type I IFN response induces several host factors capable of restricting specific steps of HIV-1 replication at late stages of its life cycle in the absence of viral countermeasures; portraying a sophisticated and redundant defense mechanism (reviewed in [5]). Recently, TRIM22 has been shown to be a correlate of HIV-1 infection [34] and can block HIV-1 Gag particle production by altering intracellular trafficking of Gag to the plasma membrane and by inhibiting transcription from the HIV-1 long terminal repeat [7,35]. Rhesus TRIM5α has also been shown to degrade intracellular HIV-1 Gag polyproteins [36,37]. ISG15 has been shown to interfere with late stages of HIV-1 Gag budding by disrupting interactions between Gag and Tsg101 [31] and between VPS4 and LIP5 [29,30], interactions that are necessary for membrane scission and virus release. In the absence of HIV-1 Vpu, the host factor BST-2 (Tetherin) retains mature virions on the cell surface that are then subsequently endocytosed [38,39].

The HERC5-induced post-translational modification of Gag is a potentially novel innate defense mechanism against HIV-1. It is unknown whether the modification of Gag is a prerequisite or consequence of restriction. Data presented by Durfee and colleagues (2010) show that HERC5 co-translationally modifies newly synthesized proteins with ISG15. Although we showed here that HERC5 interacts with Gag and co-localizes with Gag in cells, additional experiments are needed to determine if HERC5 post-translationally modifies newly made Gag polyproteins and if this modification directly interferes with Gag assembly at the plasma membrane. It is currently unclear how or why HERC5 accumulates more in a region near the plasma membrane in the presence of Gag and the conjugation system. It is possible that some HERC5 associates with newly synthesized Gag on the way to the plasma membrane. However, it is also possible that HERC5 is recruited to regions of Gag assembly by other mechanisms. Further experiments are required to determine how and why the localization of some HERC5 protein changes in the presence of Gag.

The assembly and budding processes of HIV-1 Gag particles are relatively well understood (reviewed in [23,40-45]), however much remains to be known about how the initial HIV-1 Gag bud forms and how the bud morphs into the "lollipop" structure. It has been suggested that energetic requirements for membrane distortion, which is a prerequisite for budding, occurs through the higher-order oligomerization of Gag molecules and is mediated through interactions between the interaction-domain of Gag. It is possible that HERC5 reduces Gag-Gag interactions below a threshold required to complete budding. HERC5-induced modification of a small fraction of Gag monomers may inhibit higher-order oligomerization and/or the recruitment of cellular factors due to steric hindrance of an attached ISG15 molecule. Alternatively, HERC5 could disrupt interactions with factors that manipulate membrane curvature and initiate or propagate formation of the bud. Such interactions have been observed between endophilin-2 and MLV Gag, and HIV-1 Gag and actin [46,47]. Further experiments will be needed to address these and other possibilities.

Consistent with three previous studies [29-31], we were able to show that expression of ISG15 together with its E1 and E2 enzymes (in the absence of HERC5 expression) restricted Gag particle production without a reduction in intracellular Gag protein. Our data, together with data obtained by Okumura A., et al. (2006), Pincetic A., et al. (2010) and Kuang Z. et al. (2011), suggest that HERC5 restriction is distinct from the anti-HIV-1 activities of ISG15-only expression in that HERC5 induces the modification of Gag, whereas ISG15 alone does not. Moreover, HERC5 induces an arrest at an early step of assembly, whereas the expression of ISG15 alone causes an arrest at a late stage of budding/release where predominantly immature virions accumulate on the cell surface in doublet pairs or in chains. Interestingly, the phenotype resulting from the expression of ISG15 alone resembles that observed with HIV-1 p6 mutants where the authors reported that these virions accumulated on the surface in doublet pairs or in chains and were largely immature [48]. The HIV-1 p6 protein regulates the final abscission step of nascent virions from the cell surface and accomplishes this primarily through the recruitment of Tsg101 and other members of the ESCRT complexes. The expression of ISG15 alone has been shown to interfere both with the recruitment of Tsg101 to p6 [31] and the interaction of Vps4 and LIP5, which are needed to promote the formation of the ESCRT-III-Vps4 double-hexamer complex required for membrane scission and virion release [29]. It is possible that in the absence of HERC5, another restrictive E3 ligase (yet to be identified) can be utilized, or that the E2-ISG15 complex can ISGylate substrate proteins in an E3 ligase-independent manner.

The ability of HERC5 to target viral proteins is not limited to HIV-1. We showed here that HERC5 blocked Gag particle production of an evolutionarily divergent retrovirus MLV. Interestingly, we found that HERC5 did not modify MLV Gag with ISG15 to appreciable levels compared to HIV-1 Gag and that HERC5 expression led to a substantial accumulation of intracellular MLV Gag protein. The reason for this difference is unclear, but could be due to differences in the rates of de-ISGylation and/or protein turnover. HERC5 E3 ligase activity has also been shown to block the antiviral function of the influenza A NS1 protein and interfere with the infectivity of human papillomavirus 16 pseudoviruses [20,21]. Given that HERC5 is present in several vertebrates, it will be interesting to learn more about the spectrum of pathogens that can be targeted by HERC5 and the mechanisms underlying its restriction.

Our analysis of transcriptional profiling data of patient samples from various stages of infection and disease progression revealed that HERC5 expression is significantly increased in viremic patients (eg. acute, chronic, and AIDS). Given our data presented here that HERC5 inhibits HIV-1 particle production, one standing question is why HERC5 is not sufficient to suppress HIV-1 infection in patients who succumb to AIDS? One possibility is that these patients express non-functional HERC5 variants. Since we showed that the HERC5-C994A mutant failed to inhibit HIV-1 particle production, it is plausible that polymorphisms in the *herc5 *gene that perturb the E3 ligase activity, or other polymorphisms affecting activity of the HERC5 protein, impact disease progression. For example, an insertion/deletion polymorphism (rs34457268) has been identified in humans that leads to a translational frameshift and the production of a truncated HERC5 protein lacking the Cys994 active site residue.

A small percentage of HIV-1 infected patients remain asymptomatic for more than 10 years and maintain high CD4 cell counts without antiretroviral therapy. There are now several lines of evidence to suggest that these HIV-1 controllers are still infected with pathogenic virus, indicating that host factors of the innate and adaptive immune responses may play an important role in limiting HIV-1 replication and disease progression. Intriguingly, our analysis revealed that HERC5 expression is significantly increased in the elite controllers of the Vigneault et al. (2011) dataset, patients who have stable CD4^+ ^T cell counts for > 10 years without any clinical signs of disease progression. Notably, gene transcripts known to be involved in intrinsic cellular defense against retroviruses, such as members of the *TRIM *and *APOBEC *gene families, BST2/tetherin, and cyclophilin A, were not expressed differently between the elite controllers and the reference patients [33]. This begs the question of whether HERC5 is one factor, in addition to others, that can impact disease progression in these patients. Future work is needed to determine if HERC5 fits with the classical description of a cellular restriction factor, whether clinical isolates of HIV-1 possess HERC5 countermeasures, and whether *herc5 *gene polymorphisms impact disease progression.

## Methods

### Ethics Statement

Informed consent was obtained from all subjects according to the ethics protocol #16682E, approved by The University of Western Ontario Research Ethics Board for Health Sciences Research Involving Human Subjects (HSREB).

### Cells and Cell Lines

Cells were maintained in standard growth medium (Dulbecco's Modified Eagle's Medium (DMEM) for adherent cells and RPMI-1640 for suspension cells), supplemented with 10% heat-inactivated Fetal Bovine Serum (FBS), 100 U/ml Penicillin and 100 μg/ml Streptomycin) at 37°C with 5% CO_2_. Cell lines were obtained from American Type Culture Collection unless otherwise stated. The murine dendritic cell line DC2.4 was described previously [49]. HOS-CD4/CXCR4 was provided by Dr. F. Bushman (University of Pennsylvania, USA). The following reagent was obtained through the NIH AIDS Research and Reference Reagent Program, Division of AIDS, NIAID, NIH: (GHOST (3) R3/X4/R5; Cat. 3943) from Dr. Vineet N. KewalRamani and Dr. Dan R. Littman [50]. Peripheral blood mononuclear cells (PBMCs) were isolated from whole blood from healthy volunteers using a Ficoll Hypaque (Sigma) gradient according to the manufacturer's instructions.

### Plasmids, transfections and antibodies

Plasmids pUbe1L, pUbcH8, myc-tagged ISG15 (pMyc-ISG15), flag-tagged HERC5 (pHERC5) and flag-tagged HERC5-C994A (pHERC5-C994A) were kindly provided by Dr. K. Chin (Genome Institute of Singapore). The promoterless empty vector plasmid pGL3 was purchased from Promega, p3xFLAG from Sigma, and pUbp43 from Open Biosystems. pLKO.1/scrambled_shRNA _was obtained from Addgene plasmid 1864; sequence 5' cctaa ggtta agtcg ccctc gctct agcga gggcg actta acctt agg 3') [51]. pLKO.1/HERC5_shRNA _(cat. #RHS4533-NM_016323, TRCN0000004169) was obtained from Open Biosystems. The plasmid encoding histidine-tagged ISG15 (pHis-ISG15) was created from the pMyc-ISG15 template by amplifying ISG15 using PCR and the primers: forward- 5' ACG TAA GCT TAC CAT GCA TCA TCA CCA TCA CCA TGG TGA TCA AAT GGG CTG GGA CCT GAC GG 3' and reverse 5' ACG TCT CGA GTC TAG ATT AGC TCC GCC CGC CAG G 3'. The forward primer encoded a 6× His tag (underlined). The amplified product was cloned into pcDNA3.1(+) (Invitrogen) using HindIII and XbaI. The plasmid encoding codon-optimized Gag (pGag) was obtained through the NIH AIDS Research and Reference Reagent Program, Division of AIDS, NIAID, NIH from Drs. Yingying Li, Feng Gao and Beatrice H. Hahn (p96ZM651gag-opt) [52]. Plasmid encoding the replication-competent provirus HIV-1 R9 was obtained from Dr. F. Bushman (University of Pennsylvania, USA). Plasmid transfections were performed using standard calcium phosphate transfection for co-transfections of 3 or more plasmids or Lipofectamine 2000 (Invitrogen). Plasmids were co-transfected at a ratio of 10:7.5:7.5:7.5:1 for pHERC5 or pHERC5-C994A, pUbcH8, pUbe1L, pMyc-ISG15 and pGag or pR9 respectively, unless otherwise noted. The following reagents were obtained through the NIH AIDS Research and Reference Reagent Program, Division of AIDS, NIAID, NIH: HIV-1 p24 Monoclonal Antibody (183-H12-5C) from Dr. Bruce Chesebro and Kathy Wehrly [53-55]); HIV-1SF2 p24 antiserum. MLV antiserum was a generous gift from Dr. S. Ross (University of Pennsylvania, USA). Antibodies: anti-HERC5 was obtained from Abnova, anti-FLAG from Sigma, anti-ISG15 and anti-β-actin from Rockland, anti-HA from Roche, anti-myc from Santa Cruz, and anti-ribosomal protein S6 from Cell Signaling Technology.

### Quantification of infectious virus

Clarified supernatants containing virus particles were pelleted over a 20% sucrose cushion for 2 hours at 21,000 × g. Pellets were lysed for quantitative Western blot analysis or resuspended in fresh medium and used to infect GHOST(3) indicator cells. Quantification of infectious virus release using GHOST(3) indicator cells has been described previously [50].

### Reverse transcription polymerase chain reaction

Total RNA was isolated from cell lysates using the Purelink RNA Mini Kit and reverse transcribed using M-MLV reverse transcriptase according to manufacturer's instructions (Life Technologies). The cDNA was amplified by PCR using the following primers: HERC5 forward- 5' CTG GCA CTG TTT AAG AAA C 3'; HERC5 reverse- 5' TCA GCC AAA TCC TCT G 3'; HERC3 forward- 5' ATG TTA TGT TGG GGA TAT TGG 3'; HERC3 reverse- 5' TCA GGC CAA ACT AAA CCC TTC ATA G 3'; β-actin forward- 5' GGT CAT CAC CAT TGG CAA TGA GCG G 3'; β-actin reverse- 5' GGA CTC GTC ATA CTC CTG CTT GCT G 3'. Amplified DNA were separated on a 1% agarose and quantified densitometrically using ImageJ 1.43 u 64-bit version software (NIH, USA).

### Immunoprecipitation

For immunoprecipitation, cells were lysed with cold non-denaturing lysis buffer (1% (w/v) SDS, 50 mM Tris-Cl pH 7.4, 5 mM EDTA pH 8.0, 300 mM NaCl, 0.02% (w/v) sodium azide with Roche protease inhibitor) for 20 minutes. Cyanogen bromide-activated Sepharose beads (GE Health Care) were swollen in 1 mM HCl for 10 minutes followed by antibody coupling using either 5 μl of rabbit anti-p24CA or 1 μl of mouse anti-FLAG (Sigma) per 75 μl of beads in coupling buffer (0.1 M NaHCO_3 _pH 8.3 with 0.5 M NaCl) overnight at 4°C. The beads were then washed three times with coupling buffer to remove excess antibody. The beads were blocked with blocking buffer (0.1 M Tris-HCl buffer pH 8.0) for 2 hours at 4°C. The beads where then washed with 3 cycles of alternating pH (0.1 M sodium acetate pH 4 with 0.5 M NaCl; 0.1 M Tris-HCl pH 8.0 with 0.5 M NaCl). The cell lysates were added to the beads for 1 hour at 4°C. The beads were washed 8 times with non-denaturing lysis buffer and the protein was eluted with 0.5 mM NaCl.

### Western blotting

Clarified supernatants containing virus or Gag-only particles were pelleted over a 20% sucrose cushion for 2 hours at 21,000 × g. For cell lysates, cells were detached, centrifuged at 350 × g for 5 mins, washed twice with phosphate-buffered saline (PBS). Pellets were lysed with 1× RIPA buffer (50 mM Tris-HCl (pH 7.4), 150 mM NaCl, 1 mM EDTA, 1× Complete Protease Inhibitor (Roche), 1% Triton X-100, 0.1% SDS). For quantitative Western blotting, samples were mixed with 4× loading buffer (40% Glycerol, 240 mM Tris/HCl pH 6.8, 8% SDS, 0.04% bromophenol blue, and 5% beta-mercaptoethanol) to a final 1× concentration and separated on a 10% SDS-PAGE gel. Protein was transferred to FluorTransW (Pall) membrane by semi-dry transfer. Western blotting was carried out by blocking the membrane for 1 hour in Li-cor Blocking Buffer (Li-cor Biosciences) followed by an ~16 hour incubation with 1:1000 dilution of primary antibody. Detection was carried out using IRdye-labeled secondary antibody (1:20,000 for 30 mins) and the Li-cor Odyssey Detection System (Li-cor Biosciences). Densitometric analysis was performed using ImageJ 1.43 u 64-bit version software (NIH, USA).

### Protein identification by mass spectrometry

Gel slices were excised and protein subjected to trypsin digestion followed by peptide identification by MALDI-MS using the Applied Biosystems^® ^4700 Proteomics Discovery System. A search of the NCBInr viral database using Mascot identified HIV-1 Gag protein with > 5 matching peptides and scores > 50.

### Purification of histidine-ISG15 tagged proteins

Cells were cultured in 10 cm dishes and co-transfected with empty vector plasmid or pHERC5, pUbcH8, pUbe1L, pISG15 and pGag (10:7.5:7.5:7.5:2 ratio respectively) with or without pUbp43. Nickel pull down of histidine-tagged ISG15ylated proteins was completed as previously described [20]. Briefly, cells were lysed with His-lysis buffer (50 mM Tris-Cl [pH 7.4], 300 mM NaCl, 1% Triton-X 100, 10 mM imidazole, 10 mM 2-β-mercaptoethanol) with 5 mM *N*-ethylmaleimide (Sigma), 1 mM PMSF (Sigma), and a protease inhibitor mixture (Roche). Ni-NTA agarose (Qiagen) was added to clarified cell extracts and incubated overnight at 4°C. The Ni-agarose was washed 3 times in His-lysis buffer and the protein was eluted with 0.5 M imidazole.

### Confocal Immunofluorescence Microscopy

Cells were cultured in 12-well plates on 18 mm coverslips and co-transfected with pHERC5 (or pHERC5-C994A or empty vector), pUbcH8, pUbe1L, pISG15 and pGag (10:5:5.5:1 ratio respectively). Twenty-four hours post-transfection, the coverslips containing the cells were washed twice with PF buffer (1× PBS + 1% FBS), fixed for 10 minutes in 1× PBS containing 5% formaldehyde and 2% sucrose, permeabilized in 1× PBS containing 5% NP-40 and then washed twice more with PF buffer. The coverslips were incubated with primary antibodies for one hour, washed 6× with PF buffer, incubated with secondary antibodies (Alexa Fluor 546 anti-mouse or AlexaFluor 488 anti-rabbit, Invitrogen) for one hour and then washed 6× with PF buffer. Coverslips were mounted onto glass slides with ~10 μl of Vectashield mounting media with DAPI (Vector Laboratories) and then sealed with nail polish. Slides were examined using a Zeiss LSM 510 confocal fluorescence microscope and images were obtained with sequential imaging. Spatially-calibrated images were analyzed using the "Co-localization Threshold" plugin of the ImageJ 1.43 u 64-bit version software (NIH, USA) for co-localization using the method of Costes et al. (2004) for spatial intensity correlation analysis, automatic thresholding and statistical significance testing [56]. The Pearson's correlation coefficient of co-localized volumes measures the correlation between the intensities of the two labels in the co-localized voxels and is used to express the extent of co-localization where a value of 1.0 represents perfect correlation.

### Transmission Electron Microscopy

Cells were transfected with pHERC5 or pHERC5-C994A, pUbe1L, pUbcH8, pMyc-ISG15 and pR9 (10:7.5:7.5:7.5:1 ratio) using calcium phosphate. Forty-eight hours post-transfection cells were fixed in 2.5% glutaraldehyde in a 0.1 M sodium cacodylate buffer. Cells were washed 3 times in 0.1 M sodium cacodylate buffer. The cells were fixed in 1% osmium tetroxide in 0.1 M cacodylate buffer for 1 hour and then washed 3 times in 0.1 M cacodylate buffer and enrobed in 5% noble agar. Agar was washed 5 times with distilled water then stained with 2% uranyl acetate for two hours. Agar was then dehydrated with 50% ethanol for 15 minutes then dehydrated in 70% ethanol overnight at 4°C. The next day the agar was dehydrated with 85% then 95% ethanol for 15 minutes each. The agar was dehydrated in propylene oxide for 15 minutes and added to a 1:1 ratio of epon resin/propylene oxide for 3 hours followed by incubation in a 3:1 ratio of epon resin/propylene oxide overnight at 4°C. The following day, the agar was incubated in pure epon resin for 6 hours. Lastly, the samples were kept at 60°C for 2 days. Slices were cut and visualized using a Philips EM 410 equipped with high resolution 35 mm film output. Developed photos were digitized using an Epson Expression 1680 scanner.

For immunogold staining, U2OS cells were fixed in 3% paraformaldehyde, 0.025% gluteraldehyde, in 0.1 M cacodylate (CAC) buffer adjusted to pH 7.4 for two hours. Cells were then washed with 0.1 CAC buffer. Cells were later enrobed in 5% noble agar. Solidified agar and cell mixture was dehydrated in 50, 70, 85, 95, 2× 100% ethanol respectively. Samples were rotated in a 1:1 LR white resin:100% ethanol then in pure LR white. The sample was placed in a gelatine capsule and placed in an oven at 50°C for 24 hours. Samples were cut and placed on nickel grids. Grids were blocked with a 0.2 uM filtered 1% BSA PBS Buffer (10.4 mM Na_2_HPO_4_, 3.2 mM KH_2_PO_4_, 20 mM NaN_3_, 150 mM NaCl, 1% BSA, pH 7.4) and incubated at room temperature for 2 hours with 0.25 mg/ml anti-p24CA (1:20 dilution). Samples were washed and incubated at room temperature for 2 hours with secondary goat anti-mouse IgG colloidal 10 nm gold-conjugated antibody (Invitrogen cat# A31561) at a dilution of 1:30. Samples were washed with BSA-PBS buffer and then with dH_2_0. Samples were stained with 2% uranyl acetate for 5 minutes and then washed with dH_2_0.

### Statistical Analyses

GraphPad Prism v5.03 was used for all statistical analyses stated in the text. *P *values and statistical tests were stated in the text where appropriate. *P *values less than 0.05 were deemed to be significant. *Immunogold-labeling quantification: *Quantification of immunogold localization on the electron microscopic thin sections was performed as described previously [57]. Gold particles were counted for each field of view and scored as falling on one of three regions of interest: 1) HIV-1 Gag particles and plasma membrane; 2) cytoplasm and nucleus; and 3) not containing particles or cells. The plasma membrane was defined as the outer most edge of the cell to a distance 100 nm inside the cell. 100 nm was chosen since this is approximately the diameter of a mature HIV-1 virion. The resulting numerical frequency distribution (G_0_) represents the 'observed' distribution [58]. The 'expected' or 'predicted' distribution of gold particles was determined using a randomly positioned lattice of test points (P) superimposed on each field of view. This was done by viewing the 'grid' feature in Adobe Photoshop CS3 for each image. The resulting distribution of test (or 'grid') points represents that which would be expected if gold particles were scattered randomly across the cell [58]. The number of points that fell on each of the three regions of interest were scored. The expected number of gold particles (G_e_) for each region was calculated using the formula: G_e _= P*(total G_o_)/(total P). The corresponding partial *X*^2 ^for each region was calculated from the observed and expected gold counts as: *X*^2 ^= (G_o_-G_e_)^2^/G_e_. If the total *X*^2 ^value for the given degrees of freedom (df) (given by 2-1 columns × 3-1 regions) indicated that the observed and expected distributions were significantly different, the null hypothesis of no difference from random labeling was rejected. Preferentially labeled regions were identified on the basis of satisfying two criteria. First, the G_o_/G_e _was > 1 and, secondly, the corresponding partial *X*^2 ^value accounted for a substantial proportion (≥ 10%) of the total *X*^2 ^value [58].

## List of Abbreviations

HERC5: HECT domain and RCC1-like domain-containing protein 5; IFN: interferon; RLD: Regulator of Chromosome Condensation 1 (RCC1)-like domain; CS: conjugation system; RT-PCR: reverse transcription polymerase chain reaction.

## Competing interests

The authors declare that they have no competing interests.

## Authors' contributions

SB and MW conceived and designed the experiments. SB, MW, JK, CH, JT, LX, MC and GQ performed the experiments and analyzed the data. SB wrote the paper. MW, JS and SB discussed the experiments and edited the paper. All authors read and approved the final manuscript.
